# Shunt surgery in iNPH patients is cost-effective – a cost-utility analysis in the Western Sweden setting

**DOI:** 10.1186/2045-8118-12-S1-O50

**Published:** 2015-09-18

**Authors:** Mats Tullberg, Jakob Petersen, Josefine Persson, Daniel Jaraj, Kerstin Andrén, Per Hellström, Carsten Wikkelsö, Åsa Lundgren-Nilsson

**Affiliations:** 1 Hydrocephalus Research Unit, The Sahlgrenska Academy, University of Gothenburg, Sweden; 2 Gothia Forum for Clinical Research, Sahlgrenska University Hospital, Sweden

## Introduction

Idiopathic normal pressure hydrocephalus (iNPH) is a treatable but underdiagnosed condition. The number of diagnosed patients will probably increase in the future which will increase costs and challenge allocation of resources. Shunt surgery is successful in most patients and we recently reported that 86% of operated patients improved their health related quality of life (HRQOL), almost to the same level as found in the normal population. However, the cost-effectiveness of treatment for iNPH has not been reported. The aim of this study was to simulate the long-term effects and costs of shunt surgery versus no treatment in iNPH in a Swedish setting.

## Methods

The cost-utility analysis of shunt surgery was based on a decision-analytic Markov model adapted to Swedish circumstances. The effectiveness measure was quality-adjusted life years (QALYs) gained per patient. Costs were derived from the European iNPH Multicenter Study and data on costs of dementia disorders in Sweden reported by the National Board of Health and Welfare. Data from thirty-seven patients with iNPH (median age 70 years, range 50-89 years) evaluated before and six months after surgery with the EQ-5D (EuroQol Group-5 Dimension health survey) was used to calculate quality adjusted life years (QALYs). Figures of prevalence, natural course of iNPH and transition probabilities were obtained from the literature. One-way sensitivity analysis and probabilistic sensitivity analysis were carried out to investigate the robustness of the model.

## Results

The preliminary model showed that shunt surgery in iNPH resulted in a gain of 3.03 life years and 3.12 QALYs along with an incremental cost of approximately $48,021 or £32,014. The incremental cost-effectiveness ratio (ICER) was estimated to $15,550 or £10,000 per gained QALY.

## Conclusions

From the preliminary result of the simulation model, it can be concluded that shunt treatment in iNPH is cost-effective. The estimated average ICER of £10,000 per gained QALY is below the UK National Institute for Health and Care Excellence (NICE) acceptance level of £20,000 for cost-effective interventions.
